# A Review of the Lidocaine in the Perioperative Period

**DOI:** 10.3390/jpm13121699

**Published:** 2023-12-11

**Authors:** Abigail Silva, Joana Mourão, Nuno Vale

**Affiliations:** 1 PerMed Research Group, Center for Health Technology and Services Research (CINTESIS), Rua Doutor Plácido da Costa, 4200-450 Porto, Portugal; abigailsilva@outlook.pt; 2 CINTESIS@RISE, Faculty of Medicine, University of Porto, Alameda Professor Hernâni Monteiro, 4200-319 Porto, Portugal; joanamourao@med.up.pt; 3 Department of Anesthesiology, Centro Hospitalar Universitário de São João, Alameda Professor Hernâni Monteiro, 4200-319 Porto, Portugal; 4 Surgery and Physiology Department, Faculty of Medicine, University of Porto, Rua Doutor Plácido da Costa, 4200-450 Porto, Portugal; 5 Department of Community Medicine, Health Information and Decision (MEDCIDS), Faculty of Medicine, University of Porto, Rua Doutor Plácido da Costa, 4200-450 Porto, Portugal

**Keywords:** lidocaine, pain control, perioperative opioid, administration

## Abstract

This review analyzes the controversies surrounding lidocaine (LIDO), a widely recognized local anesthetic, by exploring its multifaceted effects on pain control in the perioperative setting. The article critically analyzes debates about lidocaine’s efficacy, safety, and optimal administration methods. While acknowledging its well-documented analgesic attributes, the text highlights the ongoing controversies in its application. The goal is to provide clinicians with a comprehensive understanding of the current discourse, enabling informed decisions about incorporating lidocaine into perioperative protocols. On the other hand, emphasizes the common uses of lidocaine and its potential role in personalized medicine. It discusses the medication’s versatility, including its application in anesthesia, chronic pain, and cardiovascular diseases. The text recognizes lidocaine’s widespread use in medical practice and its ability to be combined with other drugs, showcasing its adaptability for individualized treatments. Additionally, it explores the incorporation of lidocaine into hyaluronic acid injections and its impact on pharmacokinetics, signaling innovative approaches. The discussion centers on how lidocaine, within the realm of personalized medicine, can offer safer and more comfortable experiences for patients through tailored treatments.

## 1. Introduction

Lidocaine (LIDO) was discovered in the 1940s by Nils Lofgren and Bengt Lundquist and it is one of the most popular drugs. This drug is an amide anesthetic used for local anesthetic procedures. It was initially used intravenously as an antiarrhythmic agent. It was later proposed that intravenous LIDO had an analgesic effect that could potentially be beneficial in perioperative settings. Since then, LIDO has been a local anesthetic and antiarrhythmic widely used in several areas of medical practice [1,2,3,4].

**Figure 1:**
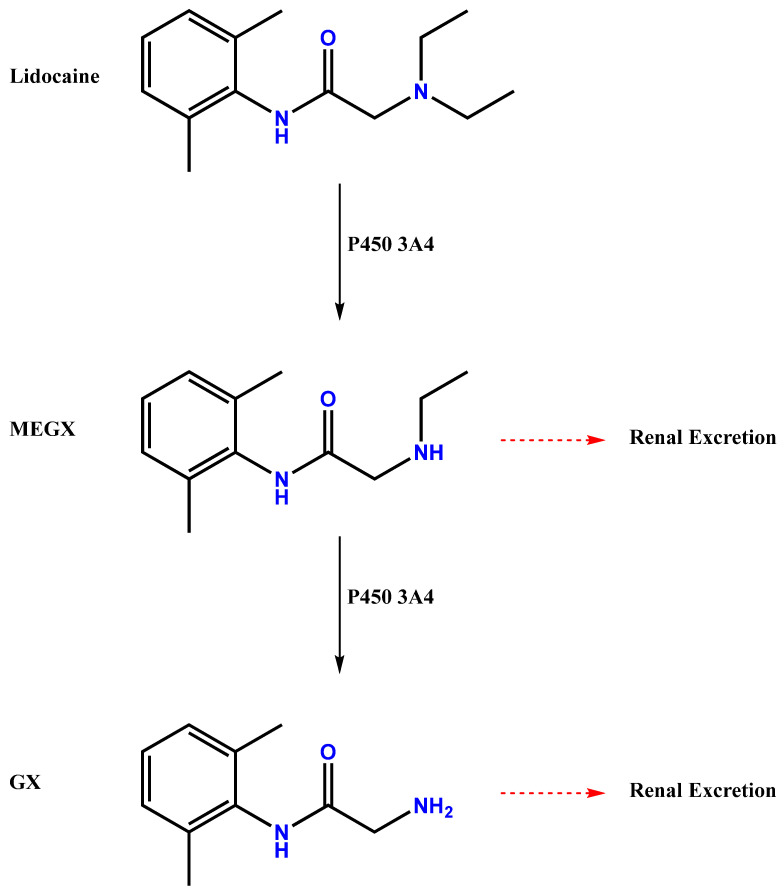
Chemical structure of Lidocaine (LIDO) and metabolism pathway.

This drug is known for its effectiveness in reducing pain and preventing cardiac arrhythmias, accounting for its wide use in healthcare. LIDO is a class Ib antiarrhythmic agent on the Vaughan–Williams classification scale. Its molecular formula is C_14_H_22_N_2_O, and its chemical structure is characterized by an aliphatic chain linked to an aromatic ring and an amine group (Figure 1). LIDO is the monocarboxylic acid amide resulting from the formal condensation of N,N-diethylglycine with 2,6-dimethylaniline [5,6]. LIDO is a white crystalline powder at room temperature; it is readily soluble in water and has a slightly bitter taste [7].

LIDO is used to relieve localized or regional pain by acting directly on the peripheral nerves. It is often used in the perioperative period. This period refers to the time interval before, during, and after a surgical procedure; during this period, it is common to administer drugs and opioids to relieve post-operative pain. Opioids are potent analgesics that can cause various side effects, including respiratory depression, bradycardia, low blood pressure, vomiting, drowsiness, pruritus, and constipation. Therefore, their use is carefully managed to provide pain relief while minimizing these side effects. LIDO, a local anesthetic, is also frequently used in the perioperative period. It is administered intravenously to treat pain in various types of operations [8,9,10].

It is often administered by injection, subcutaneous or infiltration, or as an oral gel. It can be applied for local or regional anesthesia, cardiac arrhythmia control, or chronic pain (in this type of formulation Versatis 700 mg Medicated Plaster—Patient Information Leaflet, medicines.org.uk). It is used in minor surgical procedures, dentistry, and obstetrics, where it is injected at the site of the intervention to temporarily block the conduction of pain signals to the brain. It is also used as an intravenous antiarrhythmic in emergencies, such as ventricular fibrillation and ventricular tachycardia, helping to normalize the heart rhythm. In cases of chronic pain, LIDO can be administered in the form of transdermal patches to provide localized relief over time [11,12]. When drugs are combined for anesthesia, they should contribute to the same effect and provide improved pain management. The use of one or more drugs can lead to drug interactions (DDI) or adverse drug reactions (ADR). Drugs can interact in three separate ways: pharmaceutical, pharmacodynamic, or pharmacokinetic. To decrease the severity or incidence of effects without impairing the desired effects, the drugs should be adapted to each case [13,14,15,16,17].

The dosage of LIDO varies widely depending on the application (Table 1) [6].

LIDO’s chemical structure comprises three main components, common to all local anesthetics: an aromatic ring, an intermediate bond, and a terminal amine. The essential functional groups in this molecule are amine (NH_2_) and amide (C=O), which are present in its structure. These groups play a key role in LIDO’s local anesthetic activity, interacting with the sodium channels in neuron membranes. Like LIDO, other local anesthetics interrupt nerve impulse conduction by preventing sodium ions from entering. In many cases, this occurs after these ions have diffused through the neural membrane into the axoplasm. Within the axoplasm, they access the sodium channels and prevent these channels from entering an active or “open” state. Neurons that transmit pain signals and neural messages have sodium channels responsible for propagating these signals. When LIDO is administered, it specifically blocks these sodium channels, preventing sodium ions from entering the nerve cells. This therefore inhibits the transmission of the pain signal to the central nervous system, resulting in local anesthesia, pain relief, and, in some cases, the control of cardiac arrhythmias [19,20,21,22].

Furthermore, when administered systemically, this medication exhibits remarkable anti-nociceptive characteristics, especially in the context of acute postoperative pain and chronic neuropathic pain, covering both acute and chronic pain situations. LIDO also modulates NMDA receptors, which play a role in chronic pain. By inhibiting NMDA receptors, LIDO interferes with the transmission of pain signals and can reduce central sensitization [23]. Findings from numerous preclinical studies indicate the existence of several possible molecular mechanisms operating at the cellular, subcellular, regional, and systemic levels, contributing to LIDO’s analgesic properties. These additional mechanisms deepen our understanding of the therapeutic effects of this drug in various pain conditions [22,23,24].

This drug is produced synthetically in pharmaceutical laboratories. The manufacturing process involves chemical synthesis from specific raw materials and is synthesized in a two-step process. The first step involves the reaction between 2-chloroacetyl chloride and 2,6-dimethylaniline to form an intermediate α-chloro amide. The second step involves an SN_2_ reaction between this intermediate and diethyl amine to form the final product, lidocaine [25,26]. In vivo, LIDO rapidly and extensively undergoes sequential oxidative *N*-dealkylation by cytochrome P450-3A4 in the liver to produce two active metabolites: monoethylglycylxylidide (MEGX) and glycylxylidide (GX) (Figure 1) [27].

In summary, LIDO is a drug of great importance in medicine due to its effectiveness in reducing pain and controlling cardiac arrhythmias. Its unique chemical structure and selective mechanism of action on sodium channels make it a valuable choice in various clinical applications. Research continues to explore new ways to harness its benefits, further expanding our understanding of this essential drug [6].

## 2. Initial Strategy

Lidocaine (LIDO) has proven to be an invaluable asset within hospital settings since its introduction. Its effectiveness as a local anesthetic, delivering rapid pain relief, has rendered it the preferred choice for a diverse range of medical procedures. The utilization of LIDO in hospitals varies widely, contingent upon the specific procedure and the unique needs of the patient. Its adaptability and efficacy in managing pain and facilitating various medical interventions are manifold [28,29,30,31,32].

The primary applications of LIDO in hospital settings encompass local and regional anesthesia, notably in major and minor nerve blocks [28]. LIDO injection solution can induce local numbness (anesthesia) when administered in the vicinity of a surgical site. Furthermore, it can be employed for localized anesthesia by injecting the solution near the nerves requiring interruption of conduction or into the epidural space proximate to the spinal cord [28]. Additionally, LIDO finds utility in medical procedures such as cystoscopy, proctoscopy, bladder tube insertion, endoscopy, and endotracheal intubation [29,32]. For dental procedures, including tartar cleaning, gum anesthesia before anesthetic injection, or radiography, LIDO stands as the preferred choice for treatment [29].

LIDO is also instrumental in pain relief, offering respite from the discomfort associated with conditions like sunburn, shingles, cold sores, cracked breasts, hemorrhoids, and anal fissures [29]. For the treatment of pain arising from cystitis or urethritis, LIDO gel, at a concentration of 20 mg/g, is administered by healthcare professionals by doctor-recommended dosages, particularly before procedures like cystoscopy, catheterization, bladder tube insertion, or for addressing pain stemming from cystitis and urethritis [30].

LIDO is further employed for the control of dysrhythmias, with its rapid onset of action occurring approximately one minute after intravenous injection and fifteen minutes following intramuscular injection. Its distribution swiftly extends to the surrounding tissues [28,32,33].

In the event of a heart attack, an initial dose of 1 to 1.5 mg/kg is administered (infusion rate: 25 to 50 mg/min), with the possibility of repeating the dose at 0.5 to 1 mg/kg after 5 min (maximum dose: 3 mg/kg) [31]. In general, the maximum safe dose per body weight can be considered to be 3 mg/kg, or 7 mg/kg when using preparations with epinephrine [6].

LIDO plays a pivotal role before procedures such as endotracheal intubation, endoscopy, or defibrillation [20]. In minor surgeries, including dental operations, superficial suturing, and the insertion or removal of objects from the skin, LIDO serves as a local anesthetic [28]. Furthermore, it offers pain relief during examination procedures and instrumentation such as cystoscopy, proctoscopy, and endotracheal intubations [29]. It is also indicated for the temporary alleviation of pain associated with skin injuries or minor burns [29].

Finally, LIDO’s versatility extends to major surgical interventions, encompassing open abdominal, laparoscopic abdominal, prostate, breast, thoracic, ambulatory, multilevel spine, cardiac, laparoscopic renal, abdominal hysterectomy, and hip arthroplasty procedures (Table 2).

There are two primary methods for delivering a parenteral drug solution directly into a patient’s vein: intravenous bolus and intravenous infusion. An intravenous bolus entails the swift injection of a solution into a vein, typically completed within seconds or a few minutes. Conversely, an intravenous (IV) infusion involves the gradual administration of the drug solution over extended periods. Short IV infusions may last anywhere from 10 to 20 min, while long infusions can extend over several hours or even days [33]. In these surgeries, lido helps to reduce pain, opioid consumption, and nausea, leading to a shorter recovery time in the hospital [34].

## 3. Pharmacology and Mechanism of Action

LIDO undergoes metabolism in the liver through *N*-dealkylation and hydroxylation by microsomal P-450 enzymes [35].

**Figure 2:**
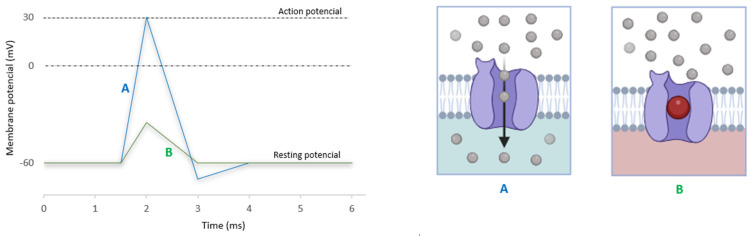
Representation of LIDO’s blocks voltage-gated sodium channels to prevent neuronal depolarization and block the transmission of pain. (**A**): Sodium channel triggered action potential. (**B**): Sodium channel action potential being inhibited by LIDO. Adapted from [37] and developed in BioRender on 15 September 2023.

It acts primarily by blocking voltage-gated sodium channels (VGSCs) on the inner surface of nerve cell membranes, with the uncharged form diffusing through neural sheaths into the axoplasm before ionizing by combining hydrogen ions. This resulting cation binds reversibly to sodium channels from within, keeping them open and preventing nerve depolarization (Figure 2) [36]. LIDO’s lower pKa of 7.7 means that approximately 25% of its molecules remain un-ionized at a physiological pH of 7.4, allowing them to enter nerve cells more rapidly than local anesthetics with higher pKa values [6].

Regarding its pharmacokinetics, LIDO is 65% protein-bound to albumin and alpha1-acid glycoprotein in plasma, resulting in a medium duration of action compared to other local anesthetics. Its lower lipid solubility limits its overall potency. The volume of distribution is 0.7 to 1.5 L/kg, and hepatic enzymes metabolize it into both active and inactive metabolites.

LIDO is approximately 60–80% protein-bound when administered intravenously, mostly to α-1-acidic glycoprotein. It crosses the blood-brain barrier through passive diffusion and can exist in ionized or un-ionized forms, with 25% remaining un-ionized at a physiological pH of 7.4.

The major metabolic pathway in the liver involves N-dealkylation, mainly by CYP3A4, producing the pharmacologically active metabolite monoethylglycinexylidide (MEGX) and further metabolites like glycinexylidide (GX) and N-ethylglycine (EG). MEGX is 80% as potent as the parent drug, while GX is nearly ineffective. LIDO is primarily excreted in the urine (90–95% as metabolites and 5–10% as unchanged drug), with an elimination half-life of 90 to 120 min in most patients, potentially prolonged in patients with hepatic insufficiency or congestive heart failure [38,39]. LIDO’s mechanism of action involves sodium channel blockade, influenced by its pKa value and the presence of inflammation. Its pharmacokinetics include protein binding, low lipid solubility, and hepatic metabolism, with potential variations in patients with specific medical conditions [40].

Excessive blood levels of LIDO can induce toxicity, as patients experience changes in cardiac output and rhythm, total peripheral resistance, mean arterial pressure, and also changes in consciousness. These alterations are linked to several factors, including the blockage of autonomic fibers, the direct depressive impact of the local anesthetic on various aspects of the cardiovascular system, and the potential beta-adrenergic receptor stimulation when epinephrine is present. Typically, this results in a modest hypotensive effect when adhering to recommended dosages [19].

The primary mechanism of these cardiac effects is associated with LIDO’s principal action, which occurs when it binds to and blocks sodium channels. This action inhibits the ionic movements necessary for initiating and conducting electrical impulses required for muscle contraction. In cardiac myocytes, LIDO can potentially hinder or slow the rise of cardiac action potentials and the accompanying contractions, leading to potential outcomes like hypotension, bradycardia, myocardial depression, cardiac arrhythmias, and, in severe cases, cardiac arrest or circulatory collapse [5,41].

This swift onset of action is evident within about one minute after intravenous injection and approximately fifteen minutes following intramuscular injection. After administration, LIDO quickly spreads through the surrounding tissues, providing anesthetic effects lasting approximately ten to twenty minutes when given intravenously and about sixty to ninety minutes after intramuscular injection [42].

However, the efficacy of LIDO may be compromised in the presence of inflammation. This reduction in effectiveness may stem from factors such as acidosis, which reduces the quantity of unionized LIDO molecules, a more rapid decline in LIDO concentration due to increased blood flow, or potentially the increased production of inflammatory mediators like peroxynitrite, which directly affects sodium channels [4,33,34,35,36,37,38,39,40,41,42,43,44,45].

LIDO is easily absorbed by damaged skin and mucous membranes but is poorly absorbed through intact skin. It quickly enters the bloodstream after absorption from the upper airway, tracheobronchial tree, and alveoli. While it is well absorbed across the gastrointestinal tract, its oral bioavailability is only about 35% due to significant first-pass metabolism. Injection into tissues results in rapid absorption, influenced by tissue vascularity and the presence of fat capable of binding LIDO. The concentration of LIDO in the blood is influenced by factors such as the rate of absorption from the injection site, tissue distribution, and metabolism/excretion rates [5]. The site of injection, dosage, and pharmacological profile also affect systemic absorption. The highest blood concentrations occur after the injection into the mucosa, which blocks the intercostal nerve, followed by the lumbar epidural space, the brachial plexus site, and finally the subcutaneous tissue. Regardless of the site, the total injected dose primarily determines absorption rate and blood levels. A linear relationship exists between the injected LIDO amount and peak anesthetic blood levels. LIDO hydrochloride is completely absorbed following parenteral administration, with absorption affected by lipid solubility and the presence of vasoconstrictor agents. The highest blood levels are achieved after intercostal nerve block, while subcutaneous administration results in the lowest blood levels. LIDO crosses both the blood-brain and placental barriers, likely via passive diffusion [35,36,37,38,39,40,41,42,43,44,45,46,47,48].

LIDO is primarily and rapidly metabolized by the liver. Metabolites and unchanged drugs are excreted by the kidneys. Biotransformation includes processes like oxidative N-dealkylation, ring hydroxylation, cleavage of the amide linkage, and conjugation. N-dealkylation, a major metabolic pathway, yields metabolites monoethylglycinexylidide and glycinexylidide. These metabolites have pharmacological/toxicological actions similar to but less potent than LIDO HCl. Approximately 90% of administered LIDO HCl is excreted in the form of various metabolites, with less than 10% excreted unchanged. The primary metabolite in urine is a conjugate of 4-hydroxy-2,6-dimethylaniline [49].

Unchanged LIDO and its metabolites are primarily excreted via the kidneys, with less than 5% appearing in the urine unchanged. Renal clearance is inversely related to LIDO’s protein binding affinity and urine pH, suggesting non-ionic diffusion for excretion.

The excretion of unchanged LIDO and its metabolites primarily occurs via the kidney, with less than 5% appearing unchanged in urine. Renal clearance is inversely related to LIDO’s protein binding affinity and urine pH, suggesting non-ionic diffusion for excretion. The mean systemic clearance for intravenously administered LIDO in adults was approximately 0.64 ± 0.18 L/min. Liver dysfunction can significantly prolong LIDO’s half-life, possibly twofold or more.

LIDO has a volume of distribution ranging from 0.7 to 1.5 L/kg. It is distributed throughout the total body water and tissues, with higher concentrations in highly perfused organs and a significant presence in skeletal muscle due to mass.

Approximately 60 to 80% of LIDO is bound to plasma proteins. The extent of binding depends on plasma alpha-1-acid glycoprotein concentration, contributing to LIDO’s medium duration of action compared to other local anesthetic agents.

The elimination half-life of LIDO hydrochloride after intravenous bolus injection is typically 1.5 to 2.0 h. Liver dysfunction can significantly prolong LIDO’s half-life, possibly twofold or more [6,50].

When a local anesthetic binds to sodium channels, it disrupts the flow of sodium ions (Na+), inhibiting action potential generation and transmission. Local anesthetics prefer binding to sodium channels when they are in the open or inactivated state. Consequently, the onset of local anesthetic effects is faster in neurons that fire rapidly. This effect extends to various ion channels, including sodium channels, potassium channels, transient receptor potential channels, hyperpolarization-activated cyclic nucleotide-gated channels, acid-sensing ion channels, G-protein-coupled receptors, acetylcholine receptors, glutamate receptors, serotonin receptors, opioid receptors, purine receptors, toll-like receptors, GABA receptors, and glycine receptors [23].

Voltage-gated sodium channels (VGSCs) are crucial for electrical excitability and the generation and propagation of action potentials in excitable membranes, forming the molecular basis of nervous system function, including sensory transmission and nociceptive signaling. Six of the nine VGSC isoforms are expressed in somatosensory primary afferent neurons, such as the dorsal root ganglion (DRG), and are involved in the propagation of physiological neuropathic and inflammatory pain. Intravenous lidocaine has been shown to suppress ectopic discharges in injured DRG or peripheral nerves, with the Nav1.8 channel being about five times more sensitive to lidocaine than the Nav1.7 channel or other channel subtypes. Lidocaine shows a frequency-dependent block, meaning block intensity increases at higher action potential firing frequencies. This may explain why systemic lidocaine blocks ectopic activity in injured nerves, while normal nociception remains unchanged at the same concentration. In the spinal cord, lidocaine at concentrations of 43–64 mM decreased the response to dorsal root C-fibre stimulation and suppressed the prolonged ventral root potentials. Intravenous lidocaine suppressed the noxious-stimulus-evoked activity in dorsal-horn wide-dynamic-range neurons, while spontaneous potentials and activity induced by non-noxious stimuli remained unaltered. The clinical effects of lidocaine in chronic pain patients far outlast plasma concentrations, pointing to a supra-spinal mechanism, involving other targets than sodium channels [23,51,52,53].

Potassium channels play many physiological roles in excitable cells, including regulating resting potential, action potential firing rate, and repolarization. Certain types of potassium channels are involved in pain modulation and are potential future targets in pain therapy. Lidocaine has been shown to block various types of potassium channels. For example, it blocks the voltage-insensitive ‘flicker’ K+ channel in demyelinated Xenopus laevis nerve fibers, KV in sciatic nerve fibers, and rat brain KV1.1 channel and isolated DRG. Lidocaine also inhibits both KV1.1 and KV3.1 channels in a concentration-dependent manner. K2P channels mediate background or ‘leak’ K+ currents and are essential regulators of the resting membrane potential and excitability. Lidocaine has been shown to inhibit several K2P channels, including TASK and TREK1. Lidocaine also induces fast and reversible inhibition of three channels of the Kir2.x subfamily (Kir2.1, Kir2.2, and Kir2.3), albeit at concentrations above clinically relevant levels [23,54,55].

Voltage-gated Ca^2+^ channels (VGCCs) regulate many physiological processes, including neurotransmitter release. Several types of VGCCs exist and are categorized according to their voltage sensitivity. High-voltage-activated calcium channels include the L-type (CaV1.1-CaV1.4), P/Q-type (CaV2.1), N-type (CaV2.2), and R-type (CaV2.3) channels. Under pathological conditions, like nerve injuries, dysregulation of VGCC is associated with increased pain sensation. Because of their significant influence on neurotransmitter release, they are considered potential targets for chronic pain therapy. Lidocaine inhibits neuronal VGCCs in isolated nerve cell bodies of frog DRG at concentrations in the millimolar range, and also in mammalian DRG neurons. However, much higher doses (~100-fold) of lidocaine are needed to inhibit VGCCs compared with VGSCs, limiting the degree of Ca^2+^ channel blockade by lidocaine [23,56,57].

Transient receptor potential (TRP) ion channels are key in detecting environmental stimuli and are expressed in nociceptors that convey thermal, chemical, and mechanical stimuli. Lidocaine has been observed to affect TRPV1 and TRPA1 receptors, potentially contributing to pain upon injection of lidocaine, and neuroinflammatory and neurotoxic processes. However, these effects occur at concentrations much higher than plasma concentrations observed after intravenous lidocaine [23,58,59].

Hyperpolarization-activated cyclic nucleotide-gated (HCN) channels play a significant role in neuronal excitability by regulating cell activity. Lidocaine has been shown to inhibit HCN channels, potentially reducing excitability in dorsal-horn neurons [23,60].

Acid-sensing ion channels (ASICs) are voltage-insensitive cation-selective ion channels that sense protons and are activated by extracellular acidosis. They are involved in nociceptive circuits. Lidocaine has been shown to block ASIC currents at high concentrations [23,61].

G-protein-coupled receptors (GPCRs) are the largest family of membrane signaling proteins and can adopt numerous conformations. They play a significant role in pain states, and their expression and subtype composition may change considerably. Lidocaine has been found to interfere with muscarinic, thromboxane A2 (TXA2), and lysophosphatidic signaling, primarily via the Gaq subunit [23,62].

Acetylcholine (ACh) receptors are ligand-gated receptors activated by the neurotransmitter, ACh. The nicotinic ACh receptor (nAChR) is an ion channel found in the CNS, autonomic ganglia, and neuromuscular junction, while the muscarinic ACh receptor (mAChR) belongs to the GPCR family. Both receptor types have been implicated in pain transmission. Lidocaine has been shown to inhibit m1 and m3 muscarinic receptors, with m1 muscarinic receptor signaling being inhibited more potently. Prolonged exposure of m1 and m3 receptors to lidocaine resulted in a biphasic time course with initial inhibition, followed by enhanced signaling. Both the muscle-type nAChR and the neuronal nAChR are also targets of lidocaine [23,63].

Glutamate, the major excitatory neurotransmitter in the CNS, is heavily involved in nociception. Lidocaine has been shown to inhibit NMDA-induced currents and activation of NMDA receptors, albeit at high concentrations. Lidocaine can also inhibit glutamate release from nerve terminals and enhance the activity of the glutamate transporter, EAAT3 [23].

Serotonin (5-hydroxytryptamine [5-HT]) mediates dual actions in pain modulation, with both pro- and antinociceptive effects via various 5-HT receptor subtypes. Lidocaine has been shown to inhibit 5-HT-induced currents by HT3 receptors at relatively high concentrations. In rats with neuropathic pain, the intrathecal injection of the serotonin receptor antagonists suppressed the pain-relieving effect of lidocaine, presumably releasing the descending pain-inhibitory systems [23,64].

Opioid receptors are GPCRs without a Gaq-subunit and lidocaine inhibits GPCRs via Gaq subunits, so there is no direct interaction between lidocaine and recombinant m-, k-, and d-opioid receptors. However, co-administration of opioids and lidocaine synergistically potentiates anti-nociception [23].

Purinergic receptors participate in multiple pathways, including neuropathic pain. Lidocaine predominantly inhibited P2X7 subunits whilst leaving P2X3 and P2X4 almost unaffected. Considering that P2X7 receptors are expressed in microglia, the contribution of P2X7 inhibition to the anti-hyperalgesic effect of lidocaine might be attributable to interference with microglia-associated inflammatory mechanisms [65].

Toll-like receptors (TLRs) play a pivotal role in the innate immune system by recognizing pathogens and initiating an immune response. Lidocaine has been shown to inhibit the activation of TLR4, and subsequently also nuclear factor (NF)-kB and mitogen-activated protein kinases (MAPKs) in lipopolysaccharide (LPS)-stimulated murine macrophages. Lidocaine also reduced organ failure in rats with LPS-induced sepsis by down-regulating TLR4 [23,66].

GABA, an important inhibitory neurotransmitter in the CNS, plays a pivotal role in the processing of nociceptive information. Lidocaine has been shown to interfere with GABAergic signaling. It can inhibit GABA release and GABA-induced Cl-currents, but also potentiate GABA-mediated Cl- currents by inhibiting GABA uptake. This might explain the antinociceptive effects of lidocaine, given that other GABA uptake inhibitors have well-documented beneficial effects in chronic pain. Lidocaine can also alleviate neuropathic pain by interfering with GABAergic neurotransmission involving the descending inhibitory system. However, the role of the GABAergic system in lidocaine-induced antinociception is complex and requires more research [23,67].

Glycine receptors are ionotropic receptors that conduct chloride currents. Lidocaine or its metabolites have been suggested to have a glycine-like action in the CNS. The anti-hyperalgesic effect of intravenous lidocaine in rats was reversed by intrathecal pretreatment with strychnine and with modulators of the glycine B site of the NMDA receptor, suggesting that part of the antinociceptive effect of systemic lidocaine might be attributable to glycine-like actions of lidocaine or its metabolites. Lidocaine metabolites, MEGX and EG, were shown to act as inhibitors of the glycine transporter, GlyT1, at clinically relevant concentrations. Systemic administration of EG significantly ameliorated hyperalgesia and allodynia in both inflammatory and neuropathic pain, suggesting that systemic lidocaine may produce anti-hyperalgesia through its metabolite EG by inhibition of spinal GlyT1, and hence, facilitating inhibitory neurotransmission [23,68].

Nitric oxide (NO) is a gaseous signaling molecule regulated by the enzyme NO synthase (NOS). Lidocaine has been shown to dose-dependently inhibit NO production and inhibit inducible NOS (iNOS), possibly involving VGSCs. It also inhibits the TNF-a-induced activation of endothelial NOS (eNOS) in lung microvascular endothelial cells, preventing NO production and further propagation of inflammatory signaling and microRNAs (miRNAs) serve as important regulators of gene expression by translational inhibition or mRNA degradation and play a role in the development and maintenance of chronic pain. Lidocaine has been shown to interact with miRNAs in adipose stem cells, up-regulating four miRNAs (miR-9a*, miR-29a, miR296-5p, and miR-373). It also down-regulates miR-34a/c and let-7b and up-regulates miR-493 in the context of local anesthetic neurotoxicity. However, it is unclear whether the regulation of certain miRNAs contributes to the clinical analgesic effects of lidocaine [23,69].

## 4. Adverse Effects

Alongside its positive effects, LIDO also causes unwanted adverse effects. Most of these side effects occur when plasma concentrations of LIDO rise to toxic levels. Smaller amounts can still lead to side effects and toxicity if administered intravascularly [8,70].

LIDO is believed to have more neurotoxicity compared to other local anesthetics, particularly when applied directly to nervous tissue. The use of highly concentrated LIDO (2.5 to 5%) for spinal anesthesia is associated with a higher incidence of transient radicular irritation syndrome, a self-limiting painful condition affecting the lower body [71,72,73,74].

Anaphylactic reactions to LIDO are possible but rare. Methemoglobinemia can occur due to LIDO metabolism to O-toluidine, especially with high doses or in patients taking other medications that can precipitate methemoglobinemia. LIDO should not be used as an antiarrhythmic if the dysrhythmia may be secondary to local anesthetic toxicity [6,72,74].

LIDO preparations containing epinephrine can have cardiovascular effects, even in small amounts. Monitoring of hemodynamics is recommended before and during the use of solutions containing vasopressors, but also this could serve as an advisory for inadvertent injection on intravascular, particularly when there are concerns about the patient’s cardiovascular status. Symptoms of LIDO overdose and systemic toxicity involve central nervous system toxicity and can progress in severity. Initial symptoms may include circumoral paresthesia, tongue numbness, light-headedness, hyperacusis, and tinnitus. More serious symptoms include visual disturbances, muscular tremors, muscle twitching, and generalized convulsions. It is essential not to mistake these symptoms for neurotic behavior. In severe cases, unconsciousness and grand mal convulsions may occur. Hypoxia and hypercapnia can develop rapidly due to increased muscular activity, interfering with normal respiration. Acidosis can exacerbate the toxic effects of local anesthetics. Severe cases may exhibit hypotension, bradycardia, arrhythmia, and cardiac arrest, which can be fatal [11,73,74].

Administration of LIDO should cease immediately if toxicity is suspected. In cases of cardiorespiratory collapse, the rules of advanced life support must be followed. Some of the side effects of LIDO use may be heart problems, such as arrhythmias; breathing problems, such as difficulty breathing, irregular breathing, or cough; neurological disorders, such as convulsions, dizziness, loss of consciousness, and confusion; allergic reactions such as hives, itching, and swelling; visual and auditory symptoms, such as blurred vision, ringing in the ears, and hearing loss; and other symptoms such as chest pain, fever, sweating and weakness, bluish-colored lips, fingernails, or palms blurred, or even puffiness or swelling of the eyelids or around the eyes, face, lips, or tongue, and vomiting [75].

Adverse reactions to the administration of this drug are similar to those observed with other local anesthetics of the amide class. The most serious adverse reactions tend to be systemic. Generally, these adverse reactions are dose-related and can result from high blood levels caused by excessive dosage, rapid absorption, or inadvertent intravascular injection. LIDO can also cause a series of adverse effects in the different systems of the body. In the cardiovascular system, it can lead to decreased heart rate, low blood pressure, cardiovascular collapse, and even cardiac arrest. In the nervous system, the effects can include dizziness, headaches, drowsiness, and convulsions. In the gastrointestinal tract, it can cause vomiting, nausea, loss of bowel control, and difficulty swallowing. Some individuals may experience nervousness, confusion, and agitation. When used on the skin, irritation, burning sensations, and even skin problems can occur at or around the plaster application site, which can include redness, rash, itching, burning, dermatitis, and small blisters. More rarely, skin lesions and sores can occur, as well as open wounds, severe allergic reactions, and allergies. In the genitourinary system, it can result in loss of bladder control and sexual function. In addition, some individuals have reported tinnitus, a sensation of heat, and edema. In the respiratory system, it can cause respiratory depression and bronchospasm [75].

Studies carried out to assess the adverse effects of LIDO used as a therapy for myocardial infarction or suspected myocardial infarction recorded more adverse effects in patients receiving lidocaine (51%) than in those receiving placebo (16%; *p* < 0.0001), and patients receiving LIDO had more adverse effects in the first 12 h than in the second 12 h (50% vs. 19%; *p* < 0.001). Patients without a heart attack who received lidocaine had more adverse effects than patients with a heart attack who received the same dose (64% vs. 39%; *p* = 0.002). This study showed that the adverse effects of prophylactic lidocaine have been underestimated in the past and may cancel out its antiarrhythmic efficacy [74]. LIDO is thought to be more neurotoxic than other local anesthetics [6].

## 5. Combinations with LIDO

LIDO is often used in medical settings as a local anesthetic, either alone or in combination with other drugs (Table 3), depending on the purpose of the treatment [72].

LIDO with Epinephrine: LIDO is often combined with epinephrine (adrenaline). Epinephrine is a vasoconstrictor that reduces local blood flow when co-administered with LIDO. This prolongs the duration of the local anesthetic action and reduces the systemic absorption of LIDO. It is common in surgical and dental procedures.

LIDO with Corticosteroids: In some situations, LIDO is combined with corticosteroids. Corticosteroids have anti-inflammatory properties and, when used with LIDO, can relieve pain and inflammation in conditions such as arthritis or bursitis.

LIDO with Other Local Anesthetics: Depending on the purpose of the procedure, LIDO can be combined with other local anesthetics to achieve a longer or more comprehensive anesthetic effect.

LIDO with Arrhythmia Medications: LIDO is also used as an intravenous antiarrhythmic in cases of severe cardiac arrhythmias. Depending on the situation, it can be used in conjunction with other antiarrhythmic drugs, such as amiodarone.

LIDO with Benzodiazepines: As both drugs can have sedative effects, using them together could potentially increase the risk of excessive sedation or other side effects. Benzodiazepines can interact with other medicines that increase their sedative effect, like LIDO.

LIDO with Clonidine: Clonidine is a frequently used adjuvant to local anesthetics. The addition of clonidine to local anesthetics for peripheral nerve blocks extends the duration of analgesia and motor blockade by around 2 h. However, it increases the risk of hypotension, fainting, and sedation. When these two drugs are used together, they produce an additive inhibitory effect [71,84,85,86,87,88,89].

When used with other drugs, LIDO can cause adverse effects, resulting in pharmacological interactions between these two or more drugs, the drug-drug interaction (Table 4). These interactions can affect the efficacy of LIDO as well as patient safety. Understanding these interactions is crucial to ensuring proper treatment and avoiding potential health risks.

## 6. Interests with LIDO

LIDO was first synthesized in 1943 and began to be marketed in 1949. Although the exact date when it began to be used in hospitals is not documented, it likely began to be used shortly after it was marketed, given its effectiveness as a local anesthetic. LIDO was introduced into hospitals to provide fast and effective local anesthesia during medical and surgical procedures. Therefore, LIDO was introduced to hospitals as an effective and safe local anesthetic for a variety of medical procedures. Its ability to provide fast and effective local anesthesia has made it a popular choice for use in hospital settings [99]. Hyperbaric 5% LIDO has been available for intrathecal use since 1954. Although the initial studies of this drug concluded that it was a safe drug for short procedures, its use has been questioned ever since; in a study made for continuous spinal anesthesia, cases of cauda equina syndrome have been reported. As a result, it has been suggested that LIDO not be used for spinal anesthesia. Patients showed a higher rate of back pain extending to the thighs and legs when administered with LIDO when compared to other drugs or general anesthesia. In addition, there were seven cases of cauda equina syndrome after a single injection of hyperbaric LIDO, which is a condition that results in permanent disability. This occurred in patients of various ages and with doses ranging from 60 mg to 120 mg. Considering the potential risk of permanent neurological damage and the availability of safer alternatives, the continued use of intrathecal LIDO in the 21st century was questioned [100]. Later, a reasonable body of evidence, along with extensive clinical experience, suggests that intravenous LIDO can have a useful pain-relieving effect and is worth consideration [101]. Nowadays LIDO is frequently used in hospitals, as it possesses analgesic properties and exhibits antihyperalgesic and anti-inflammatory characteristics, making it a valuable adjunct in general anesthesia, administering an initial bolus followed by a continuous LIDO infusion offers distinct analgesic advantages. LIDO plays a pivotal role in opioid-sparing or opioid-free anesthesia approaches, markedly enhancing postoperative outcomes and patient satisfaction. By adhering to recommended protocols, LIDO can be employed with a high degree of safety and efficacy; LIDO is one of the major drugs for opioid-reduced anesthesia or opioid-free anesthesia procedures [102,103].

## 7. Future of LIDO in Personalized Medicine

LIDO, a widely used local anesthetic in medical practice, has incredible potential to play a significant role in personalized medicine in the future. Its versatility and the growing understanding of its pharmacological effects make it a promising candidate for optimizing individual treatments and improving health.

LIDO is a medication routinely used by doctors and healthcare professionals worldwide. It is employed to anesthetize specific areas of the body before surgical, dental, or diagnostic procedures. Its applications have expanded to include the treatment of chronic pain and heart diseases, making it one of the most versatile active ingredients in medicine.

A notable feature of LIDO is its ability to be combined with other drugs. For instance, LIDO is often paired with epinephrine (adrenaline). Epinephrine is a vasoconstrictor that reduces local blood flow when used alongside LIDO. This extends the anesthetic effect, reduces systemic LIDO absorption, and lowers the risk of toxicity. These combinations can be tailored to individual patient needs.

Personalized medicine aims to optimize treatment based on each patient’s unique characteristics. LIDO can play a crucial role in achieving this by enabling more precise control of anesthesia based on factors such as pain sensitivity, metabolism, and the patient’s medical history. Instead of administering a standardized dose, physicians can adjust the concentration and quantity of LIDO based on the patient’s response, resulting in more effective and comfortable anesthesia.

One of the most intriguing aspects of LIDO’s potential in personalized medicine lies in its ability to explore and utilize its side effects, which are typically considered undesirable. LIDO’s side effects can serve as markers of an individual’s susceptibility. People prone to these side effects can receive personalized treatment that considers these predisposing factors, leading to safer and more effective use.

Furthermore, instances of side effects can be analyzed to better understand how LIDO interacts with the body. This could lead to advancements in identifying patients at high risk of side effects and developing strategies to minimize them.

Personalized medicine allows LIDO treatment to be tailored to the patient’s response. This may include the selection of other local anesthetics and optimizing dosage to balance effectiveness and safety. Additionally, LIDO is more than just a tool for customizing anesthesia, it also plays a significant role in identifying individual sensitivities to medications and predicting how patients will respond to other treatments. For example, individuals who exhibit unusual reactions to LIDO may have genetic or metabolic characteristics that affect the efficacy of other drugs.

Personalized medicine has greatly benefited from advances in technology and genomics. Genetic tests provide valuable insights into how patients respond to LIDO and other drugs. For instance, mutations in genes related to metabolism can impact the rate at which LIDO is cleared from the body. Healthcare professionals can use this data to make informed decisions about LIDO use, tailoring treatment according to an individual’s genetic profile. This enhances treatment efficacy and reduces the risk of side effects.

In addition, the use of LIDO and other individualized approaches must be based on solid scientific evidence and ethical practices to improve patient health and well-being. The future of personalized medicine, centered on LIDO, promises a more precise and effective approach to medicine. There have already been some studies on lidocaine in personalized medicine and new forms of its application. One of these studies investigated the incorporation of lidocaine into hyaluronic acid (LHA) injections and its impact on the pharmacokinetic characteristics of lidocaine and its active metabolites, monoethylglycylxylidide (MEGX) and glycylxylidide (GX). To this end, a new bioanalytical method was developed to simultaneously determine lidocaine, MEGX, and GX in rat plasma. The plasma concentrations of these substances were determined after subcutaneous injection of 0.3% lidocaine solution or LHA with 0.3–1% lidocaine in rats. The data obtained was used to develop a pharmacokinetic model for the injection of LHA. In addition, a pharmacokinetic model was built to predict the concentrations of lidocaine and its two metabolites simultaneously, using the data collected from the rats [104]. So, with the new approaches to drugs nowadays, patients can expect treatment tailored to their individual needs, ensuring a safer and more comfortable experience during medical procedures. In addition, continued research into LIDO and its potential in personalized medicine could lead to major advances in understanding our body’s response to drugs and anesthetics. This deeper understanding could open doors to new, personalized treatments in several medical areas. One important aspect that needs to be researched is how LIDO can be indicated for pediatric patients, the elderly, and people with certain medical conditions. Individualized treatment for these groups could significantly improve quality of life and patient safety. Furthermore, LIDO is not limited to the field of local anesthesia. It is also being investigated for its potential in the treatment of chronic pain, especially when administered via nerve block technology. Personalized medicine can be a valuable tool in adapting these therapies to the specific needs of chronic pain patients, considering factors such as pain sensitivity and an individual’s response to LIDO. Personalized medicine can play an important role in selecting the ideal pharmacological partner when LIDO is used in combination with other agents. Individual customization of these combinations can improve treatment efficacy and reduce the risk of unwanted side effects. In summary, LIDO has incredible versatility in medicine and offers great opportunities for personalized medicine. Its ability to individualize anesthesia, identify individual susceptibility, and adapt treatment to specific genetic profiles makes it a promising candidate for the future of medicine. As advances in this area continue, we can expect more effective, safer, and more individualized treatments that meet the unique needs of each individual.

## Figures and Tables

**Table 1:** This table provides all LIDO-containing medications approved by the FDA, along with their concentrations, their compositions, and concentrations [5,6,18].

Drug Name	Drugs in Its Composition	Application Method	Concentration (%)
Lidocaine	Lidocaine	Topical	5
Lidocaine and Prilocaine	Lidocaine; Prilocaine	Topical	2.5; 2.5
Lidoderm	Lidocaine	Topical	5
Oraqix	Lidocaine; Prilocaine	Periodontal	2.5; 2.5
Pliaglis	Lidocaine; Tetracaine	Topical	7; 7
Versatis	Lidocaine	Topical	700 mg
Xylocaine	Lidocaine	Topical	5
Xylocaine	Lidocaine hydrochloride	Injectable	0.5 to 2
Xylocaine	Lidocaine hydrochloride	Topical	2
Zingo	Lidocaine; Hydrochloride	Intradermal	0.5 mg
ZTLIDO	Lidocaine	Topical	1.8

**Table 2:** A scheme that describes LIDO’s infusion and bolus that are used in specific clinical surgeries [34].

Type of Surgery	Bolus (mg/kg)	Infusion (mg kg^−1^ h^−1^)
Open abdominal	1.5–2	1.5–5
Laparoscopic abdominal	1.5	1–2
Prostate	1.5	1.5–2
Breast	1.5	1.5–2
Ambulatory	1.5	2
Multilevel spine	No bolus	2
Cardiac	1–1.5	0.033
Laparoscopic renal	1.5	2 then 1.3 PO
Abdominal hysterectomy	1.5	2–3
Hip arthroplasty	1.5	1.5
Thoracic	No bolus	0.033

**Table 3:** A scheme that describes LIDO’s combinations used in the perioperative period [75,76,77,78,79,80,81,82,83].

Drugs Combination with LIDO	Effect	Administration
Propofol	The combination resulted in a 50% reduction in propofol dose. Fatigue and pain felt after the surgery.	IV injection
Epinephrine	Increases the duration of action of lidocaine, prolonging its effect.	Injection
Antiarrhythmic (ex: Amiodarone)	The risk or severity of generalized seizure and bradycardia can be increased when amiodarone is combined with lidocaine.	IV, Oral
Cimetidine	Cimetidine reduces the systemic clearance of lidocaine. The absorption of lidocaine by erythrocytes is decreased by cimetidine. There is an interaction between lidocaine and cimetidine in terms of increasing lidocaine serum levels, as this may be mediated by cimetidine’s inhibition of the H2 receptor.	IV, Oral
Anti-hypertensive (e.g.: Beta-blockers)	This can result in bradycardia (slow heart rate) and hypotension (low blood pressure). Beta-blockers can potentiate the effects of lidocaine, increasing the duration of its action.	IV, Oral
Anticoagulants (e.g.: Warfarin)	The metabolism of warfarin can be decreased when combined with lidocaine.	Oral

**Table 4:** A scheme that describes LIDO’s interactions between drugs [90,91,92,93,94,95,96,97,98].

Drugs Combination with LIDO	Effect
Propafenone	The serum concentration of lidocaine can be increased when it is combined with propafenone.
Saquinavir	The metabolism of lidocaine can be decreased when combined with saquinavir, also saquinavir can increase the blood levels of lidocaine to dangerous levels and cause an irregular heart rhythm.
Darunavir/Cobicistat	The metabolism of lidocaine can be decreased when these two drugs are used together. Also, these drugs can affect the rhythm of your heart and cause cardiovascular problems.
Atazanavir	Using these drugs can increase the effects of lidocaine and affect the rhythm of the heart. Irregular heartbeat, chest tightness, blurred vision, or nausea can be adverse effects.
